# Detection of Bone Metastases on Bone Scans through Image Classification with Contrastive Learning

**DOI:** 10.3390/jpm11121248

**Published:** 2021-11-24

**Authors:** Te-Chun Hsieh, Chiung-Wei Liao, Yung-Chi Lai, Kin-Man Law, Pak-Ki Chan, Chia-Hung Kao

**Affiliations:** 1 Department of Nuclear Medicine and PET Center, China Medical University Hospital, Taichung 404, Taiwan; d10119@mail.cmuh.org.tw (T.-C.H.); D24364@mail.cmuh.org.tw (C.-W.L.); d24690@mail.cmuh.org.tw (Y.-C.L.); 2 Department of Biomedical Imaging and Radiological Science, China Medical University, Taichung 404, Taiwan; 3 Center of Augmented Intelligence in Healthcare, China Medical University Hospital, Taichung 404, Taiwan; T35269@mail.cmuh.org.tw (K.-M.L.); T33768@mail.cmuh.org.tw (P.-K.C.); 4 Department of Computer Science and Engineering, National Chung Hsing University, Taichung 402, Taiwan; 5 Graduate Institute of Biomedical Sciences, School of Medicine, College of Medicine, China Medical University, Taichung 404, Taiwan; 6 Department of Bioinformatics and Medical Engineering, Asia University, Taichung 413, Taiwan

**Keywords:** contrastive learning, convolutional neural network, bone scan, deep learning

## Abstract

Patients with bone metastases have poor prognoses. A bone scan is a commonly applied diagnostic tool for this condition. However, its accuracy is limited by the nonspecific character of radiopharmaceutical accumulation, which indicates all-cause bone remodeling. The current study evaluated deep learning techniques to improve the efficacy of bone metastasis detection on bone scans, retrospectively examining 19,041 patients aged 22 to 92 years who underwent bone scans between May 2011 and December 2019. We developed several functional imaging binary classification deep learning algorithms suitable for bone scans. The presence or absence of bone metastases as a reference standard was determined through a review of image reports by nuclear medicine physicians. Classification was conducted with convolutional neural network-based (CNN-based), residual neural network (ResNet), and densely connected convolutional networks (DenseNet) models, with and without contrastive learning. Each set of bone scans contained anterior and posterior images with resolutions of 1024 × 256 pixels. A total of 37,427 image sets were analyzed. The overall performance of all models improved with contrastive learning. The accuracy, precision, recall, F1 score, area under the receiver operating characteristic curve, and negative predictive value (NPV) for the optimal model were 0.961, 0.878, 0.599, 0.712, 0.92 and 0.965, respectively. In particular, the high NPV may help physicians safely exclude bone metastases, decreasing physician workload, and improving patient care.

## 1. Introduction

With increasing lifespans, cancers have become the leading cause of death and constitute a crucial health-care issue globally. The key factor in terms of therapeutic outcome and prognosis remains the stage of cancer at diagnosis. Advanced cancers are usually accompanied by metastases to distant organs, most often in the lungs, liver, and bones [1,2]. Approximately 5% of all cancer patients have bone metastases at initial diagnosis [3,4,5]. Prognoses vary but are generally poor for patients with bone metastases. The longest median survival time, approximately 2 years, is found with breast, prostate, and thyroid cancers and the shortest median survival time, less than 3 months, is seen with many cancers of the digestive system [3].

The current methods for diagnosing bone metastases are mainly based on noninvasive diagnostic imaging, such as plain radiography, computed tomography (CT), magnetic resonance imaging (MRI), positron emission tomography (PET), and bone scan, with bone scan being the most commonly used because of the routine whole-body scan procedure and high sensitivity to bone lesions. However, the bone scan employs ^99m^Tc-labeled bisphosphonates (e.g., methylene diphosphonate [MDP]), which can accumulate at sites with increased bone remodeling activity, but this accumulation is not sufficiently specific to indicate malignant tumor cells. Therefore, the efficacy of bone scans for bone metastases is compromised by its poor specificity [6,7,8]. Furthermore, bone scans usually include only a planar scan, small unsuspected lesions that may appear in a three-dimensional (3D) representation may be overlooked in such two-dimensional (2D) scans with overlapping skeletal structures. However, advanced 3D diagnostic imaging, such as CT, MRI, PET, and even additional single-photon emission CT of bone scan, is not always feasible because of the high cost and long acquisition time and the logistics of acquiring positron-emitting agents. However, emergent artificial intelligence techniques may provide another means of overcoming the problem of planar scanning.

**Figure 1:**
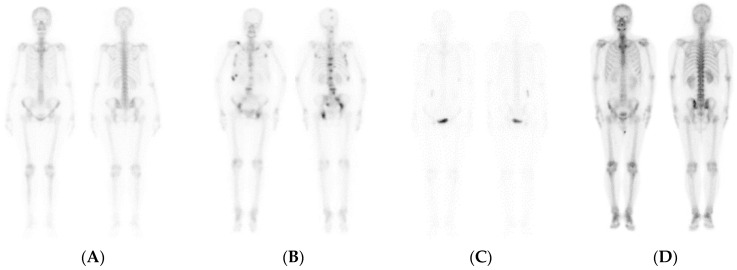
Examples of bone scans. (**A**) Normal bone scan; (**B**) multiple bone metastases present; (**C**) presenting residual urine; (**D**) presenting with high drug intake.

The bone scan, similar to other functional imaging techniques, usually has a lower spatial resolution than structural imaging methods, such as plain radiography, CT, or MRI and bone scans exhibit great variation in image quality because of individual physical and metabolic differences [9]. To overcome these drawbacks, researchers have investigated deep learning algorithms for medical image analysis [10,11,12], but such algorithms have rarely been applied to functional images. Previous studies have employed deep learning algorithms for the detection of bone metastases in prostate cancer [13,14]. For example, Papandrianos et al. used convolutional neural network (CNN) algorithms to perform functional bone scintillation image analysis [15]. Furthermore, Cook et al. explored the use of a CNN to classify whole-body bone scans of prostate cancer metastases [8]. A simpler and more effective CNN model was proposed and compared with well-known model architectures such as ResNet50, VGG16, and Xception. The accuracy of the Cook et al. proposed model on a test set was 89%. Taken together, the aforementioned results demonstrate that deep learning algorithms can be effectively applied to functional imaging classification [16]. Despite the success of CNN models in classifying bone metastases, such models face many challenges: (1) despite the images being well contrasted (Figure 1A,B), they lack accuracy in classifying tumors, infections, trauma, and arthritis; (2) CNN models are mostly trained through supervised learning, leading to annotation problems; (3) residual urine (low contrast, Figure 1C) or excessive drug intake (high contrast, Figure 1D) may lead to poor image contrast, making classification difficult. Furthermore, current CNN models still require experts to classify and label images.

Contrastive representation learning (CRL) has made considerable advances in feature learning and pretraining for computer vision [1,17]. Chaitanya et al. used contrastive learning to segment a small number of tagged medical images [18]. They proposed a new contrast method for learning the local structural similarity of an image by using contrast loss. High benchmark performance was achieved in a limited marker environment and in combination with data enhancement techniques.

This paper introduces a contrastive learning approach that improves CNN model (1) classification, (2) contrast, and (3) annotation for bone scans for binary classification (i.e., detection) of bone metastases. We compared the performance of various models using well-known CNN architectures (including DenseNet121 and ResNet50) before and after the introduction of CRL.

## 2. Materials and Methods

### 2.1. Literature Review

#### 2.1.1. CNNs

Most of the CNN architecture consists of convolutional, fully connected, and pooling layers. Its purpose is to extract features from input sources and superimpose more complex features from low-level features to perform classification tasks. The architectures of the CNN models used in this study are presented as follows.

#### 2.1.2. Model I: CNN-based

**Figure 2:**
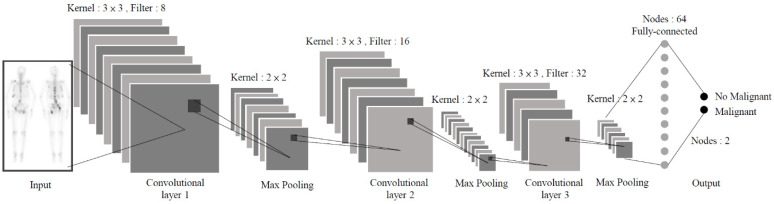
CNN-based architecture flowchart.

The CNN-based model employs the architecture proposed by the University of Thessaly and Center for Research and Technology Hellas for bone scan classification [16]. It is a deep network architecture consisting of one input layer, three convolutional and pooling layers, one flat layer, two fully connected layers, and one output layer (see Figure 2 for details). Filters are used to extract image features for classification and the network weights are updated through gradient descent and back propagation to allow the model to converge. According to experimental results, this framework is effective in classifying bone metastases.

#### 2.1.3. Model II: ResNet

The ResNet architecture was published by Ho et al. in 2015 [19]. ResNet networks are based on the VGG19 network and use residual learning to solve the degradation problem of deep learning networks by adding a residual unit to the shortcut connection. In addition, the residual network facilitates network architecture optimization and can improve the accuracy of deep networks. Using the ImageNet dataset, Ho et al. evaluated a residual network with 8 times more (152) layers than VGG, but with fewer parameters. This architecture achieved an error of 3.57% on the ImageNet test set, winning first place in the 2015 ImageNet Large Scale Visual Recognition Challenge.

#### 2.1.4. Model III: DenseNet

The DenseNet architecture was published in 2016 [20]. The authors proposed a radical dense connection mechanism in which the feature maps of all the preceding layers are used as inputs to a particular layer and the feature maps of that layer are then used as inputs for all subsequent layers. This improves the efficiency of weight transfer across the network, with each layer obtaining a reduced gradient from the loss function (i.e., feature reuse) and helps to reduce the effect of gradient disappearance on deep networks and the number of parameters. DenseNet is considerably more effective at most tasks than other advanced techniques are, requiring less computation to achieve high performance.

#### 2.1.5. CRL

**Figure 3:**
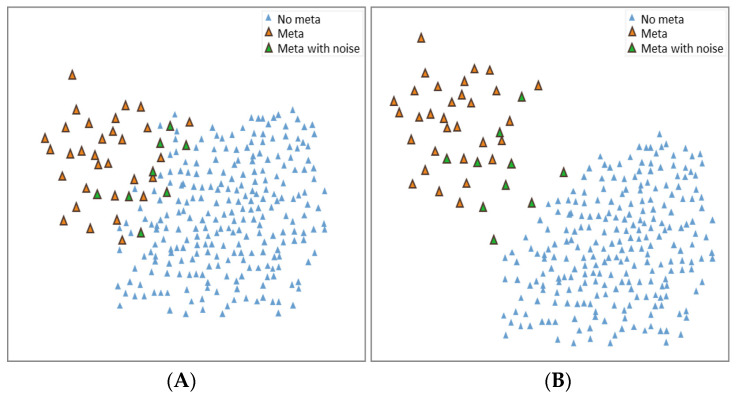
Contrast representation learning (CRL) diagram; (**A**) original data distribution, (**B**) similar distribution of data after study.

CRL is a novel approach to learning representation, allowing models to learn to distinguish similar or dissimilar images by effectively exploiting the semantic relationships between groups of samples and mining higher-level information from input images [21]. Rather than learning one feature vector at a time from a single data sample, contrastive learning involves comparing multiple samples, learning basic representations by simultaneously maximizing the consistency between different versions or views of the same image and using contrastive learning to reduce the differences. When a comparison target is used to update the parameters of the neural network, the representations of corresponding views attract each other and the representations of noncorresponding views repel each other. Thus, by contrasting positive and negative samples, the representations of the positive samples are brought together and the representations of the negative samples are distanced within a particular dimensional space (see Figure 3 for details). Contrastive learning is a simple yet powerful means of supervised or self-supervised learning of feature vectors. This study used the supervised contrastive (SupCon) learning method proposed by Google Labs for training [22]. This method is one of the CRL techniques that extends the self-supervised batch comparison method to a fully supervised environment, enabling a model to make effective use of labeling information. Clusters within the same class are attracted in the vector space and clusters from different classes are repelled. This approach improves the accuracy and robustness of classifiers over that of conventional supervised training. The method is easy to implement and enables stable training; it achieves higher accuracy with many data sets and model architectures and is robust to image noise and hyperparameter changes.

### 2.2. Research Materials and Methods

#### 2.2.1. CRL

**Figure 4:**
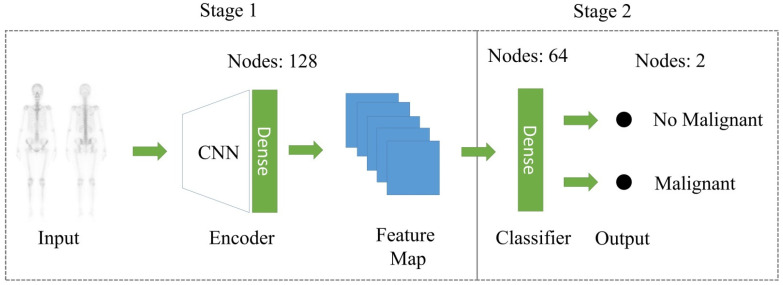
Contrastive Representation Learning Framework.

Contrastive learning is performed in two main phases (see Figure 4 for details). In the first phase, the encoder is trained to learn the representation of an input image and the loss function is learned through supervised comparison for the model to make effective use of the label information. In the second phase, the linear classifier is trained using the conventional cross-entropy loss function. Therefore, in this study, three CNN encoders, the CNN-based model, ResNet50, and DenseNet121, were used for training in the first phase of contrastive learning and then compared with supervised learning methods. According to the paper, the authors use a loss function (Equation (1)) for supervised learning that builds on the contrastive self-supervised literature by leveraging label information. Normalized embeddings from the same class are pulled closer together than embeddings from different classes. Contrastive loss consists of two aspects. First of all, the positive pair are two features obtained from the same training sample after data augmentation and the distance between these two features will become closer after training. On the contrary, the negative pair are the features from different training samples. After training, the distance between these two features will be farther. Additionally, it allows for multiple positives per anchor, thus adapting contrastive learning to the fully supervised setting.

(1) $${ℒ}_{out}^{sup}=\underset{i\in I}{\sum }{ℒ}_{out,\;i}^{sup}=\underset{i\in I}{\sum }\frac{-1}{\left|P\left(i\right)\right|}\underset{p\in P\left(i\right)}{\sum }log\frac{exp\left(z_{i}\cdot z_{p}/\tau \right)}{\sum_{a\in A\left(i\right)}exp\left(z_{i}\cdot z_{a}/\tau \right)}$$

#### 2.2.2. Experimental Data

**Figure 5:**
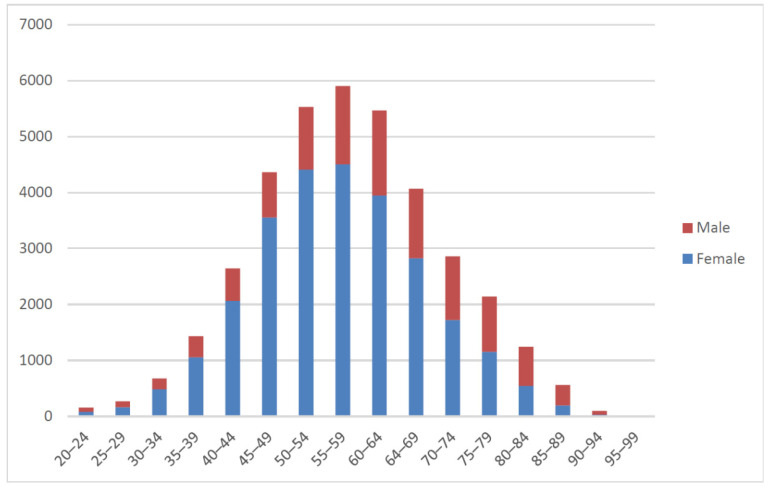
Distribution of age groups of patients.

**Figure 6:**
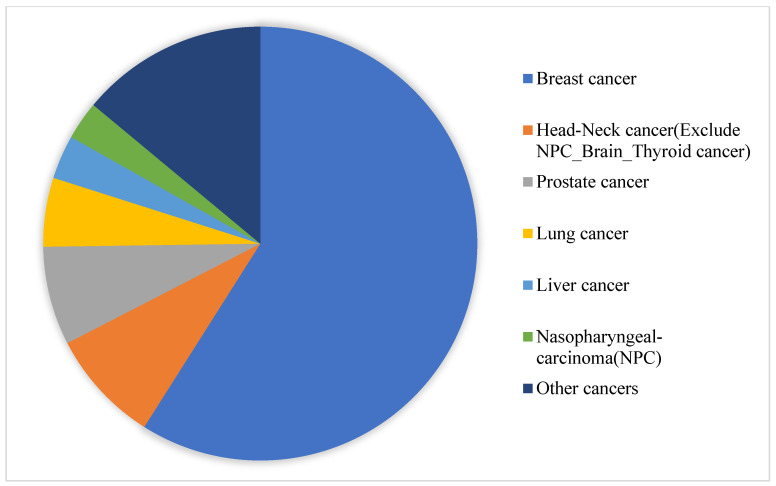
The distribution of cancer in all patients.

This study retrospectively collected 37,427 sets of images of 19,041 patients (see Figure 5) who underwent bone scans at China Medical University Hospital between May 2011 and December 2019. Routine whole-body scans were performed 2–4 h after intravenous administration of 20 mCi of ^99m^Tc-labeled MDP with a scan speed of 14–17 cm/min on either a Millennium MG, Infinia Hawkeye 4, or Discovery NM/CT 670 Pro scanner (GE Healthcare). Each set of bone scans consisted of two images, an anterior view and a posterior view, with resolutions of 1024 × 256 pixels. Of the collected images, 31,812 were used for training and 5615 were used for testing. We used DICOM raw values as model input, instead of converting to other image formats. We used pydicom, a python 3.7.0 based package, to read the pixel values of the images in the dicom file. The image shape was 1024 × 256 and had both front and back sides. We merged the two images into a shape of 1024 × 512 to facilitate the model to do a comprehensive feature interpretation of the same patient’s image. The same image was overlapped 3 times to make the shape 1024 × 512 × 3. Then, we used the average pixel value around the right thigh bone to standardize the overall image. Finally, we reduced the image shape to 256 × 256 × 3. The patients were aged between 22 and 92 years when they underwent scanning (Figure 5). The predominant cancer type (Figure 6) was breast cancer (59%), followed by head and neck cancer (9%), prostate cancer (7%), lung cancer (5%), liver cancer (3%), nasopharyngeal-carcinoma (3%), and other cancer (14%). The presence or absence of bone metastases as a reference standard was determined after a review of image reports by experienced nuclear medicine physicians and through correlation with relevant radiological studies or follow-up bone scans.

Pydicom was used for image loading and processing, Matplotlib was used for graph visualization, and Numpy was used for all mathematics and array operations. In addition, Python was used as the programming language, Keras was used for programming the models, and scikit-learn was used for data segmentation. The execution hardware was a Nvidia V100 graphics processing unit. In the model, we set 256 slices images as inputs per epoch and the overall training process ran 50 epochs with a learning rate of 0.001 by using Adam optimizer, which is the popular choice for machine learning. 

#### 2.2.3. Assessment Methods

Various metrics were used to assess the performance of the classification model on the test data. The validation metrics were accuracy (Equation (2)), sensitivity (Equation (3)), F1 score (Equation (4)), specificity (Equation (5)), precision (Equation (6)), recall (Equation (7)), NPV (Equation (8)), and area under the receiver operating characteristic (ROC) curve. In a binary classification problem, a prediction can be classified as true positive (TP), true negative (TN), false positive (FP), or false negative (FN). In our case of bone metastasis detection, a TP indicates that the label of the image is malignant and it is correctly classified. FP means that the label of the image is benign, yet it is classified as malignant. TN means that the label of the image is benign and it is classified as such. Similarly, FN means that the label of the image is malignant, yet it is classified as benign.

(2) $$Accuracy=\;\frac{TP+TN}{\begin{array}{c} \left(TP+FP+TN+FN\right) \end{array}}$$

(3) $$Sensitivity=\frac{TP}{TP+FN}$$

(4) $$F1-score=2*\frac{Recall\;*\;Precision}{Recall+Precision}$$

(5) $$Specificity=\frac{TN}{FP+TN}$$

(6) $$Precision=\frac{TP}{TP+FP}$$

(7) $$Recall=\frac{TP}{TP+FN}$$

(8) $$NPV=\frac{TN}{TN+FN}$$

#### 2.2.4. Visualization

To better understand the ability of contrastive learning to isolate different sample representations, a multidimensional representation can be transformed and remapped to a 2D space for observation through dimensionality reduction, essentially reprojecting data from a higher dimensionality to a lower dimensionality. For visualization, we employed Uniform Manifold Approximation and Projection (UMAP), a technique developed by McInnes et al. in 2018 with the primary theoretical frameworks of Riemannian geometry and algebraic topology [23]. The visualization proof preserves more of the full domain structure. In Table 1, our dataset consists of 34,386 images without metastases and 3041 images with metastases. Illustrate the distribution of training data and test data.

## 3. Results

**Figure 7:**
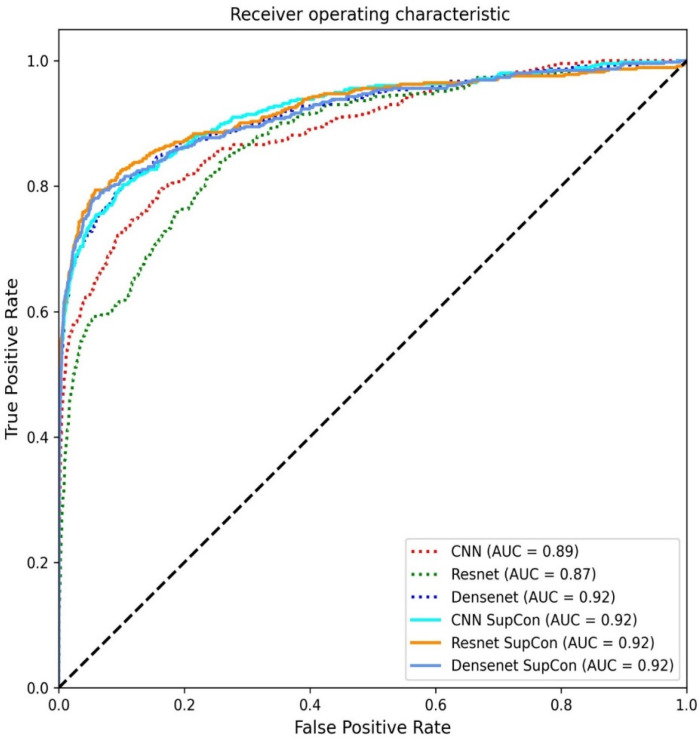
Receiver operating characteristic curve of the models in this study.

Table 2 and Figure 7 provide the evaluation metrics for different methods on the test set. For our models, all five metrics were superior to those achieved with supervised learning. This study proposes that a contrastive learning approach can improve accuracy, recall, and F1 score over conventional supervised learning. The accuracy of the CNN-based model was 94.30%, but this was improved by 1.62% with the addition of SupCon. The accuracy of the DenseNet121 model was 93.39% and improved by 2.6% with the addition of SupCon and its F1 score of DenseNet121 increased by 33.41% with SupCon. In addition, the precision of the CNN-based and DenseNet121 models increased by 27.41% and 33.33%, respectively, after the addition of SupCon. These results demonstrate that contrastive learning is effective in improving all aspects of deep learning classifiers. Table 3 presents a comparison of stratified 6-fold validation evaluation metrics for different classifiers in the test set. In this process, each of the six groups of data are selected a different subset for testing until all folds were tested. The distribution of classes in each fold closely mirrors the distribution of classes in the entire dataset.

**Figure 8:**
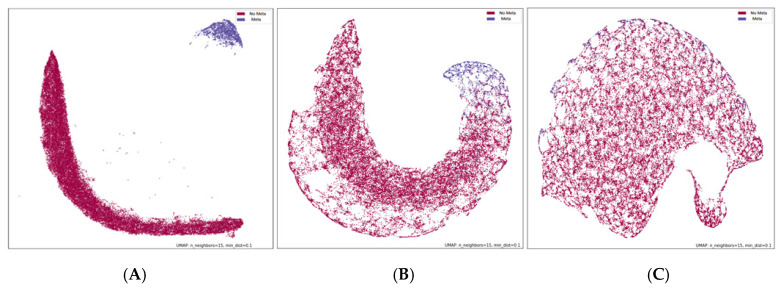
Using UMAP presenting the last layer encoder. (**A**) CNN-based; (**B**) DenseNet121; (**C**) ResNet50V2.

To facilitate understanding of contrastive learning, data visualization allows for an intuitive view of the first phase of the SupCon method. In the experiment, the last layer of the supervised contrastive learning model (including CNN-based, DenseNet121, ResNet50V2) encoder was downscaled to two dimensions through UMAP and the data distribution was presented after model training (Figure 8). As demonstrated in Figure 8, the contrastive learning CNN-based approach clearly separated each class when clustering samples, suggesting that this approach effectively extends the distance between the features of two classes, making the boundaries between clusters more visible. This approach is easy to implement and enables stable training, effectively improving the accuracy and robustness of deep learning classifiers.

## 4. Discussion

Functional medical images, such as bone scans, usually have lower spatial and contrast resolution than structural images, such as those of CT and MRI. In addition, functional images are inherently noisy and exhibited greater variation in image quality because of the individual physical and metabolic differences. Furthermore, functional medical imaging is far less common than structural medical imaging in most health-care systems. As a result, deep learning algorithms have rarely been applied to functional imaging.

Han et al. used two different CNN architectures to analyze 9113 bone scans of patients with prostate cancer. There were 2991 scans (32.8% of all scans) positive for bone metastases. The accuracy, sensitivity, specificity, PPV, NPV, and area under the receiver operating characteristic curve (AUC) for their model with better performance were 96.0%, 82.8%, 93.5%, 86.1%, 91.8%, and 0.946, respectively [24]. Papandrianos et al. selected 408 bone scans of female breast patients for analysis with CNN models, of which 221 bone scans (54.2% of all scans) were considered with bone metastases. The accuracy, sensitivity, specificity, precision, and recall for their best model were 92.5%, 94%, 92%, 93%, and 94%, respectively [15]. Zhao et al. collected 12,222 bone scans from 40 cancer types (44% of were lung cancer) and 5151 scans (42.1% of all) were regarded with bone metastases. The accuracy, sensitivity, specificity, PPV, NPV, and AUC of their deep neural network model were 93.38%, 92.64%, 93.92%, 91.75%, 94.59%, and 0.964, respectively [25]. However, the percentage of bone scans with metastases in these above studies are unusually high compared to real-world conditions. In a PET study consisting of consecutively 403 patients with histologically-proven malignant disease for initial or post-therapeutic staging, there were only 38 patients (9%) suggestive of bone metastases [26]. Another study with whole-body MRI for metastatic workup of treatment-naïve prostate cancer according to the eligible guideline of European Association of Urology, revealed the overall prevalence of bone metastases was 7% (12 of all 161 cases) in the case of newly diagnosed intermediate- and high-risk prostate cancer [27]. The prevalence of bone metastases in the above PET and MRI studies are comparable to the historical observation with cancer patients at initial diagnosis (5%) [3,4,5] and our current study (8%), which suggest that our current CNN model may be more suitable to resolve the real-world task.

Few computer-assisted systems for automatically detecting metastases on bone scans have been developed. The best known commercially available software is Bone Scan Index (BSI) [28]. BSI was developed using artificial neural networks to detect bone metastases in patients with prostate cancer through image segmentation, identifying bone areas with increased radiopharmaceutical uptake, and classifying these areas as malignant or benign lesions.

Despite its original purpose of efficiently and accurately detecting bone metastases, BSI is now used in prognostic tests for patients with high-risk prostate cancer [29]. BSI’s high FP rate limits its use in staging patients with newly diagnosed prostate cancer.

Petersen et al. used BSI to identify bone metastases in the bone scans (in Digital Imaging and Communications in Medicine format) of 342 patients with initial diagnoses of prostate cancer. They achieved a sensitivity of 93.3%, specificity of 89.3%, positive predictive value (PPV) of 57.5%, and NPV of 98.9% [30]. Wuestemann et al. enrolled 951 patients, including 406 with breast cancers and 149 with prostate cancers. They discovered that the overall efficacy in detecting bone metastases could be improved by adjusting the BSI cutoff value after an ROC analysis. The optimal results were achieved with a BSI cutoff value of 0.27%, that is, a sensitivity of 87.0%, specificity of 98.6%, PPV of 98.5%, and NPV of 87.7% [31].

This study provides a contrastive learning approach for diagnosing bone metastases on whole-body bone scans and conducted a pre- and post-importation comparison using a CNN-based model and the well-known architectures, DenseNet121 and ResNet50. The results demonstrate that contrastive learning is applicable to medical functional images and is effective in improving the accuracy of deep learning models. In addition, the method can be generalized to patients with other age distributions and provides high robustness to noisy images. Although the accuracy of each model increased with the addition of contrastive learning, some noisy image data were observed in various clusters. In future research, other deep learning architectures can be tested and greater image interpretability should be provided, with similar or higher accuracy.

Our results are comparable with those of previous BSI studies, despite the slightly lower prevalence of bone metastases in our patients. However, this did not compromise the PPV of our models, especially when contrastive learning was used. We provide a feasible technique for analyzing a large volume of images without the need to heavily preprocess individual scans for segmentation and annotation. Contrastive learning improved the overall performance of all the models tested in this study. In particular, the excellent NPV can help physicians confidently and safely rule out bone metastases. Facilitating the identification of bone scans without metastases might lessen the workload of nuclear medicine physicians and improve the overall quality of patient care.

Our study has several limitations. First, we included patients from only a single tertiary academic medical center. Multicenter studies may be needed to confirm the generalizability of our method. Second, the bone scans were performed with different scanners; however, the modality-related effect on individual images might be minimal because these scanners used similar scintillator technology and were obtained from the same manufacturer. Third, our analysis involved pooling patients with various types of cancers. Although this reflects real-world conditions, studies that analyze the performance of bone metastasis detection within cancer types are warranted to explore the efficacy and best practices of applying deep learning algorithms to such diagnosis. Fourth, the absence or presence of bone metastasis was determined by an expert’s interpretation of the bone scans rather than histological evidence. However, pathological confirmation is not always practical and may cause unnecessary harm to patients. In addition, only a few patients had bone lesions that could be differentiated by other advanced radiological modalities, such as CT or MRI. Therefore, it has been generally accepted to diagnose bone metastases by an expert’s interpretation, especially when there are multiple lesions in the axial bones that are deemed a pathognomonic feature of bone metastases.

## 5. Conclusions

Our study demonstrates that deep learning algorithms with additional contrastive learning can achieve excellent performance in detecting bone metastases Their high NPV may help physicians safely exclude bone metastases, decreasing physician workload and improving the quality of patient care.

## Figures and Tables

**Table 1:** Distribution of the image dataset of whole-body bone scan.

	No Malignant	Malignant	Total
**Train**	29,227	2585	31,812
**Test**	5159	456	5615

**Table 2:** Comparison of evaluation indicators for different classifiers in the test set.

Model	CNN	DenseNet121	ResNet50V2	CNN	DenseNet121	ResNet50V2
**Method**	Supervised Learning	Supervised Learning	Supervised Learning	Supervised Contrastive Learning	Supervised Contrastive Learning	Supervised Contrastive Learning
**Accuracy**	0.943	0.934	0.957	0.959	0.960	0.961
**Sensitivity**	0.322	0.230	0.533	0.596	0.564	0.599
**Specificity**	0.998	0.996	0.995	0.991	0.995	0.993
**Prevalence**	0.081	0.081	0.081	0.081	0.081	0.081
**Precision**	0.930	0.840	0.900	0.858	0.908	0.878
**NPV**	0.943	0.936	0.960	0.965	0.963	0.965
**F1 Score**	0.479	0.361	0.669	0.704	0.696	0.712
**TP**	147	105	243	272	257	273
**FP**	11	20	27	45	26	38
**FN**	309	351	213	184	199	183
**TN**	5148	5139	5132	5114	5133	5121

**Table 3:** Stratified 6-fold validation result on a dataset.

Model	CNN	DenseNet121	ResNet50V2	CNN	DenseNet121	ResNet50V2
**Method**	Supervised Learning	Supervised Learning	Supervised Learning	Supervised Contrastive Learning	Supervised Contrastive Learning	Supervised Contrastive Learning
**Accuracy**	0.933	0.919	0.936	0.976	0.952	0.946
**Sensitivity**	0.179	0.561	0.272	0.774	0.469	0.417
**Specificity**	1.000	0.951	0.995	0.994	0.995	0.992
**Prevalence**	0.081	0.081	0.081	0.081	0.081	0.081
**Precision**	0.975	0.695	0.576	0.923	0.888	0.694
**NPV**	0.932	0.961	0.940	0.980	0.955	0.951
**F1 Score**	0.301	0.576	0.353	0.842	0.594	0.519

## Data Availability

Not applicable.
